# Prevalence of Cytoplasmic Actin Mutations in Diffuse Large B-Cell Lymphoma and Multiple Myeloma: A Functional Assessment Based on Actin Three-Dimensional Structures

**DOI:** 10.3390/ijms21093093

**Published:** 2020-04-27

**Authors:** Laura Witjes, Marleen Van Troys, Bruno Verhasselt, Christophe Ampe

**Affiliations:** 1 Department of Biomolecular Medicine, Ghent University, Albert Baertsoenkaai 3, B-9000 Ghent, Belgium; 2 Department of Diagnostic Sciences, Ghent University Hospital, Ghent University, Corneel Heymanslaan 10, B-9000 Ghent, Belgium

**Keywords:** ACTB, ACTG1, cBioPortal, F-actin, plasma cell myeloma, lymphoid cancer, actin mutations, meta-analysis of patient data, myosin, patient cancer data

## Abstract

Mutations in actins have been linked to several developmental diseases. Their occurrence across different cancers has, however, not been investigated. Using the cBioPortal database we show that human actins are infrequently mutated in patient samples of various cancers types. Nevertheless, ranking these studies by mutational frequency suggest that some have a higher percentage of patients with *ACTB* and *ACTG1* mutations. Within studies on hematological cancers, mutations in *ACTB* and *ACTG1* are associated with lymphoid cancers since none have currently been reported in myeloid cancers. Within the different types of lymphoid cancers *ACTB* mutations are most frequent in diffuse large B-cell lymphoma (DLBCL) and *ACTG1* mutations in multiple myeloma. We mapped the ACTB and ACTG1 mutations found in these two cancer types on the 3D-structure of actin showing they are in regions important for actin polymer formation or binding to myosin. The potential effects of the mutations on actin properties imply that mutations in cytoplasmic actins deserve dedicated research in DLBCL and multiple myeloma.

## 1. Introduction

Actins form a highly conserved family of proteins [1,2]. These proteins form the central building blocks of the actin cytoskeleton which is involved in muscle contraction, cell division, adhesion and migration. These versatile cellular functions are brought about by the capacity of actins to interact with a large set of actin-binding proteins and regulating proteins that organize the polymerization of actin monomers into filaments (F-actin) [3]. Detailed 3D models of F-actin are available [4,5,6]. Humans express six actins; four of these are muscle type actins (encoded by *ACTA1, ACTA2, ACTC1* and *ACTG2*) and two are cytoplasmic forms (encoded by the *ACTB* and *ACTG1* genes).

Both *ACTB* and *ACTG1* have been found to be mutated in congenital disorders affecting development [7,8,9]. In addition, [10] reviewed dedicated studies and/or unbiased approaches monitoring altered *ACTB* expression in various cancers types and found that in general these displayed deregulated beta actin mRNA or protein expression. The mutational profiles of the two cytoplasmic actins in cancers have not been systematically investigated despite multiple “-omics” datasets on many types of cancer. Interestingly, using a novel clustering approach of rare mutations on 3D structures of proteins to distinguish non-functional passenger events from functional driver mutations, *ACTB* (and also *ACTA2*) was identified as a gene potentially involved in cancer progression [11]. A search in PubMed with the keywords ‘*ACTB*’ or ‘*ACTG1*’ and ‘mutations and cancer’ indeed yields a limited number of papers. Amongst many papers on congenital diseases also a publication on diffuse large B-cell lymphoma (DLBCL) was retrieved [12]. In addition, in three independent studies on multiple myeloma, *ACTG1* was identified as one of the most frequently mutated genes in this hematological cancer involving plasma cells [13,14,15] and it even met the criteria for potentially being a candidate driver gene in multiple myeloma. However, these studies did not compare actin mutations relative to other cancers (including other hematological cancers) nor were the mutations considered or interpreted structurally.

Hematological cancers are divided into lymphoid and myeloid cancers depending on whether cells of the tumor are of lymphoid or myeloid origin. Another way of classifying blood cancer is by their origin of location. In general, hematological cancers originating in the bone marrow are leukemias and tumors beginning in the lymphatic system are lymphomas. With over 100,000 new cases worldwide each year DLBCL is the most common hematologic malignancy [16].

Based on the initial observations from whole exome sequencing/whole genome sequencing/RNA-seq [12,13,16,17,18], we investigated whether the two cytoplasmic actins are more frequently mutated in hematological cancers compared to other cancer types and whether mutations in these two genes are recurrently observed in specific subtypes of cancers of the hematopoietic system.

By datamining cBioPortal we found that human actins are infrequently mutated in various cancers albeit some patient studies evidence a higher percentage of mutations in the cytoplasmic actins. Within hematological cancers, mutations in *ACTB* and *ACTG1* are exclusively associated with lymphoid cancers and not with myeloid cancers. Within the different types of lymphoid cancers *ACTB* mutations are for the most part associated with DLBCL. Mutations in *ACTG1* are largely associated with multiple myeloma (in cBioPortal referred to as plasma cell myeloma). Mapping the mutations on the 3D-structure of actin indicates they are not randomly distributed over the structure and are within regions required for actin polymer formation or binding to myosin. We discuss the potential functional implications with respect to the functioning of actin.

## 2. Results

### 2.1. Actin Genes Display Amplifications, Deletions And Mutations Across Several Cancers.

If the two cytoplasmic actin genes *ACTB* and *ACTG1* are more frequently mutated in hematological malignancies than sequencing screens suggest [12,13,14,15,16,17], this should be apparent from a comparison of the alteration frequency of these two genes in blood cancers relative to other cancer types. In addition, if base mutations occur in a random manner in all actins, one can expect that their alteration frequency in each of the six actin genes is similar across all cancers. This reasoning is based on the fact that the actin primary structures are evolutionarily highly conserved [1,2].

We address these issues by using data from 174 cancer studies in cBioPortal covering numerous cancers types (Table S1). From the TCGA PanCancer Atlas studies, a subset of studies available at cBioPortal, it is evident that both cytoplasmic actins are expressed and that in general beta-cytoplasmic actin mRNA is the dominant form (data available at cBioPortal, not shown). The types of alterations that occur in the six actin genes in conjunction with their frequency across all profiled cases in the 174 cancer studies were catalogued (Table 1). The cBioPortal database distinguishes between two main types of alterations: copy number alterations (CNAs, i.e., amplifications and deletions) and mutations (sense, non-sense, frame shifts, fusions). The OncoPrint of the six actin genes demonstrates that both types of alterations occur in these genes in the selected cancer studies available at cBioPortal (Figure S1). However, in general these alterations occur for all actins with low frequencies (Table 1). For the two cytoplasmic actins, CNAs are more abundant than somatic mutations. This is also the case for alterations associated with *ACTA2* and *ACTA1,* whereas for *ACTG2* and *ACTC1* CNAs are of similar frequency as somatic mutations (Table 1).

### 2.2. Cytoplasmic Actin Genes Display Mutations Across Cancer Types In A Non-Random Manner

**Figure 1:**
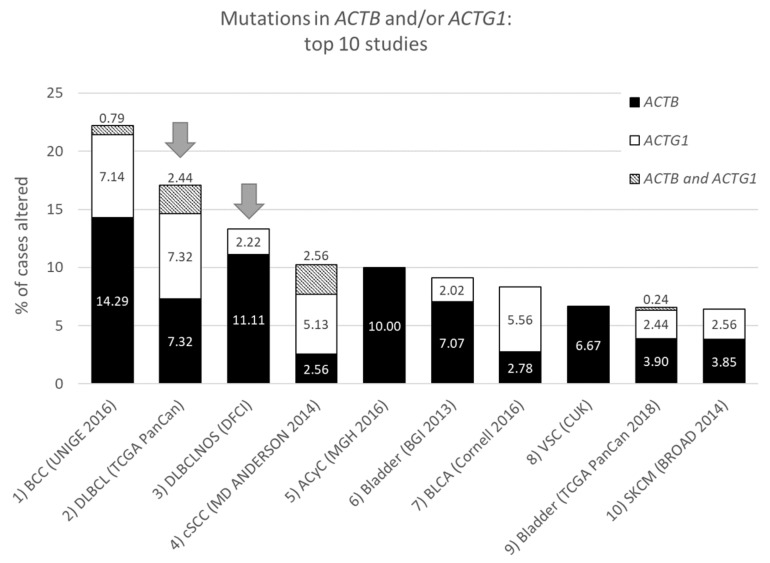
Top ten studies of cases with mutations in *ACTB* and/or *ACTG1.* Ranking is based on combined *ACTB* and *ACTG1* mutation frequency (in percent). Arrows indicate DLBCL studies at rank two and three. For full study names see Table S1. The information was derived from the Cancer Types Summary tab.

**Figure 2:**
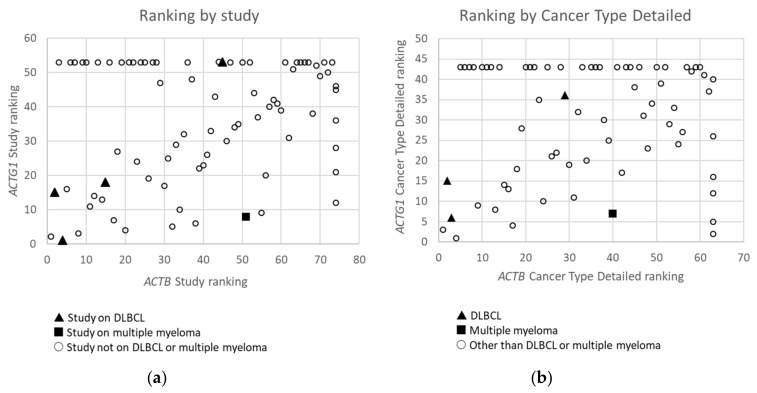
Ranking of Studies (*n* = 79) (**a**) and Cancer Types Detailed (*n* = 68) (**b**) based on frequency of mutations in *ACTB* versus these in *ACTG1* in cBioPortal. Studies or detailed cancer types in which neither mutations in *ACTB* nor *ACTG1* were found are not included. Studies or detailed cancer types in which mutations were found for *ACTB*, but not for *ACTG1* and which therefore did not receive a rank for *ACTG1*, or vice versa, have received an ‘artificial max high constant rank number’ to be able to include them in the graphs (i.e rank number set to 53 for *ACTG1* or 74 for *ACTB* in (**a**) and 43 for *ACTG1* and 63 for *ACTB* in (**b**)). Data derived from the Cancer Types Summary tab.

From the data in Table 1 it is apparent that, when all 174 cancers studies are considered, somatic mutations in actins occur with low frequency for all actins, with mutations in *ACTB* being most frequent. Inspecting only the mutation frequency data (without CNAs) for the two cytoplasmic actin genes combined, and ranking these by cancer study, reveals two skin cancer studies and two DLBCL studies within the top 5 (Table 2, Figure 1). This is largely recapitulated when considering the genes separately (Table 2). However, it is also clear that the cancer associated mutations in *ACTB* and *ACTG1* do not completely co-segregate within cancer studies (Figure 2a) nor within detailed cancer types (Figure 2b), i.e., within a cancer study or detailed cancer type a high ranking of *ACTB* does not necessarily mean a high ranking of *ACTG1* or vice versa. The low frequency of *ACTB* and *ACTG1* mutations across all cancers (Table 1) is in part explained because they are not found in many cancer types (*ACTB*: in 46 out of 75, *ACTG1*: in 29 out of 75). The extracted data underscore that the somatic mutations in *ACTB* and *ACTG1* do not occur haphazardly across cancers and that the higher frequency observed in two skin cancer and two DLBCL studies merits further investigation. We focus here on blood cancers, to which DLBCL and multiple myeloma belong, in view of existing preliminary evidence [12,13,14,15].

### 2.3. Within Hematological Cancers, Mutations in ACTB and ACTG1 are Associated with Lymphoid Cancers and not With Myeloid Cancers

**Figure 3:**
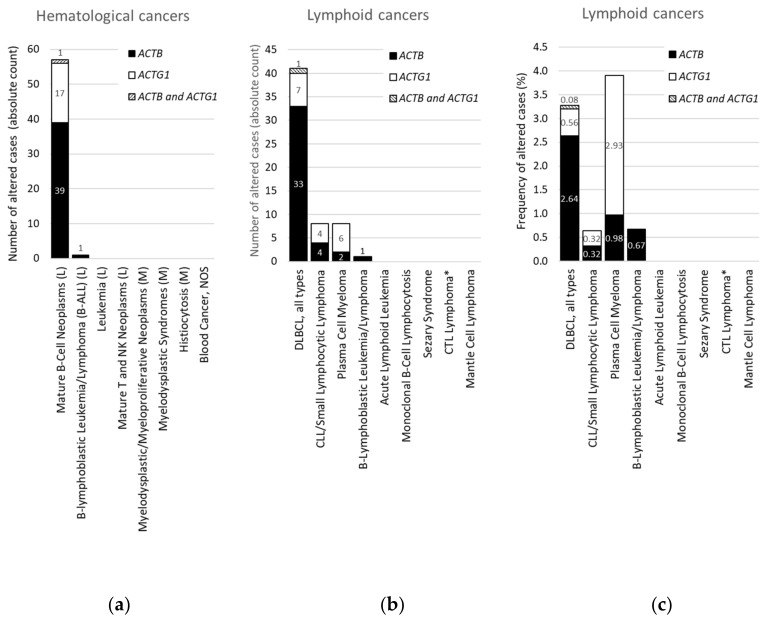
Distribution of cases with mutations in *ACTB* and/or *ACTG1* in hematological or lymphoid malignancies. (**a**) Absolute counts in hematological cancers (ranked and sorted by cancer type), (**b**) absolute counts in lymphoid cancers (ranked and sorted by cancer type detailed), (**c**) frequency in lymphoid cancers (for comparison sorted as in (**b**)). L = lymphoid cancer, M = myeloid cancer, DLBCL = diffuse large B-cell lymphoma, NOS = not otherwise specified, CLL = chronic lymphocytic leukemia, CTL = cytotoxic T-cell, plasma cell myeloma = multiple myeloma. * CTL Lymphoma = Primary Cutaneous CD8+ Aggressive Epidermotropic CTL Lymphoma. Data on ‘DLBCL, all types’ in (**b**,**c**) were originally derived from three subtypes in cBioPortal and grouped here. Information derived from the Cancer Type Summary tab.

We focused on *ACTB* and *ACTG1* in blood cancers by selecting studies on lymphoid cancers and myeloid cancers in cBioPortal (Table S2). Only a minority of these studies are profiled for CNAs (Table S2), therefore, we further concentrated on mutations. Strikingly, no mutations in *ACTB* or *ACTG1* are found in samples from patients with myeloid cancers (Table 3, Figure 3a). Indeed, focusing on the absolute counts instead of the frequencies reveals that all 58 patients with mutations in *ACTB* and/or *ACTG1* suffer from a type of lymphoid cancer (Figure 3a). This suggests that, within hematological cancers, mutations in cytoplasmic actins are associated with lymphoid malignancies rather than with cancers of the myeloid lineage.

### 2.4. For DLBCL ACTB Mutations Occur More Frequently Than ACTG1 Mutations, Whereas for Multiple Myeloma This Is the Opposite

From Figure 3a it is evident that, with the exception of a single case with an *ACTB* mutation in B-lymphoblastic leukemia/lymphoma, all hematological cancer cases that have somatic mutations in the cytoplasmic actin genes, i.e., 40 with *ACTB* and 18 with *ACTG1* mutations, are patients with mature B-cell neoplasms. To identify subtypes of lymphoid cancers potentially enriched in somatic mutations in *ACTB* and/or *ACTG1* we selected ‘cancer type detailed’ in the ‘cancer types summary’ tab after querying the lymphoid cancers in cBioPortal. Figure 3b shows that cases with *ACTB* mutations are hardly present in multiple myeloma and various lymphocytic leukemias. Of the 41 DLBCL patient samples showing cytoplasmic actin mutations 34 have one in *ACTB* whereas eight have an *ACTG1* mutation (Figure 3b). In one patient both cytoplasmic actin genes carry a mutation. More than 80% of the cases with *ACTB* mutations occur in DLBCL subtypes (34/41), whereas this is less than 20% for the two other mature B-cell neoplasms (6/41 cases). Conversely, only eight out of the 18 samples with *ACTG1* mutations are derived from DLBCL patients and approximately an equal amount from patients with plasma cell myeloma (Figure 3b). Taking into account the number of cases studied per detailed cancer type, the frequency of mutations in cytoplasmic actin genes in DLBCL reaches approximately 2.7% for *ACTB* and only 0.6% for *ACTG1* (Figure 3c). By contrast, the cBioPortal data show that 2.9% of patients with multiple myeloma have *ACTG1* mutations, whereas only 1.0% have mutations in *ACTB* (Figure 3c). Thus, *ACTB* mutations are more frequent in DLBCL patient samples relative to *ACTG1* mutations, whereas for multiple myeloma this is reversed (Figure 3b,c). In addition, out of all detailed lymphoid cancer types, patients with *ACTB* mutations are most frequent in DLBCL and patients with *ACTG1* mutations are most frequent in multiple myeloma (Figure 3c).

### 2.5. The Mutation Frequency of ACTB is Similar to that of RHOA, a Proposed Driver in DLBCL

The frequency of somatic mutations for ACTB is modest (1.2%) when considered globally across cancers (Table 1), increases to 2.7% when considered in the frame of DLCBL mutations (Figure 3c, Table 3) and amounts to approximately 10% and 11% in the DLBCL studies ranked 2^nd^ and 3^rd^ in Figure 1. From a broader perspective we compared this mutational frequency with the occurrence of mutations in major regulators of the actin cytoskeleton: small GTPases of the Rho-family. *RHOA* is recognized as driver gene in DLBCL [16] and *RAC1* is recurrently mutated or upregulated in cancers [19,20]. The data indicate that of these, only *RHOA* is frequently mutated in DLBCL with absolute counts of mutations comparable to these of *ACTB* (Table 3, Figure S2). Of interest, mutations in *ACTB* and *RHOA* do not occur simultaneously in one sample, neither do *ACTG1* and *RHOA* (Figure S2). There are fewer mutations in *ACTG1* than in *ACTB,* however, mutations in *RHOB, RHOC, RAC1, RAC2, RAC3* and *CDC42* are even more infrequent (Table 3). In addition, when compared to the frequency of *ACTB* mutation in hematological cancers the frequency of *RHOA* mutations follows an analogous pattern (Table 3). Thus, the mutational frequency of *ACTB* is similar to that of *RHOA*, a proposed driver in DLBCL.

### 2.6. The ACTB and ACTG1 Mutations in DLBCL and Multiple Myeloma are not Randomly Distributed Across the Protein’s Primary And Tertiary Sequences

Inspecting the location of the somatic mutations across all cancers (except lymphoid cancers) in the primary structure of both cytoplasmic actin proteins (with protein symbol ACTB and ACTG1) using the lollipop representations of cBioPortal reveals that some mutations are found in more than one patient and that these are spread over the entire sequence with a potential hotspot for residues G158 and E334 in ACTB (Figure S3a,b). In contrast, most of the mutations in ACTB and ACTG1 in DLCBL and multiple myeloma are concentrated in the N-terminal half of the sequence (Figure S3c–f). The ACTB and ACTG1 mutations in DLBCL and multiple myeloma are listed in Table S3. 

We included ACTG1 mutations: R62C, I64N, L65M and L110V from two additional DLBCL studies (that were not taken up in the analysis above because these two studies are partly redundant with some of the ones used). In between DLBCL and multiple myeloma, there are no common mutational sites found in ACTB or ACTG1. In addition, the only mutational site that is found in both ACTB and ACTG1 is L65 in DLBCL.

**Figure 4:**
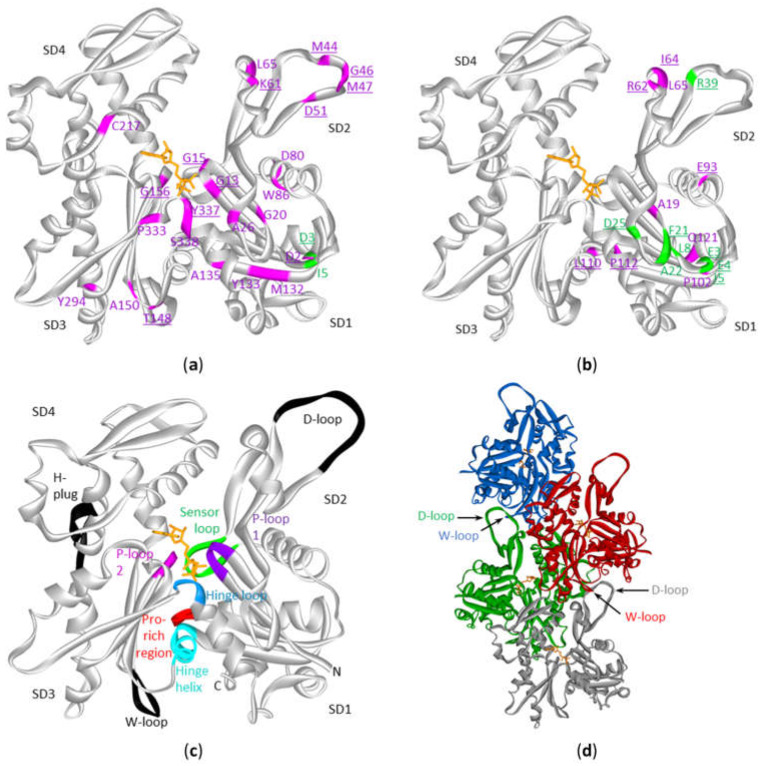
Mutations in cytoplasmic actins in DLBCL and multiple myeloma plotted on the monomeric or polymeric form of actin. Sites of mutations in ACTB (**a**) or ACTG1 (**b**) in either diffuse large B-cell lymphoma (magenta) and multiple myeloma (green) (see Table S3) with indication of subdomains (SD) 1 to 4 (amino acid residues of SD1: 1–32, 70–144, 338–375; SD2: 33–69; SD3: 145–180, 270–337; SD4: 181–269). The data are plotted on the structure of the monomeric form of ACTB (2BTF). ATP is in stick (orange). Mutation sites involved in polymer contact, ATP-binding, phosphate release (Table 4) or myosin binding (see discussion) are underlined. No analogous structure of the ACTG1 monomer is available but it is expected that the 3D structures of ACTB and ACTG1 are very similar given that their sequences only differ by 4 amino acids at the extreme N-terminus. (**c**) Structure of the actin protomer as present in the filament with indications of subdomains. Black and colored regions are important for filament formation, or binding or hydrolysis of ATP. (**d**) Four actin subunits in the F-actin structure. This is the cryo-EM structure of polymerized actin with AMPPNP (www.rscb.org/pdb: 6DJM) [6]. Each protomer is shown in a different color. The proximity of the D-loop of one protomer and the W-loop of another is indicated by arrows.

Actin is a protein with discontinuous domains, i.e., the domains are formed from two or more nonsequential segments of the primary structure. It is therefore difficult to appreciate from the primary structure (or from the lollipop diagrams) whether the mutations cluster together in the 3D-structure. This is important in view of the notion that mutations can be functional targets when they cluster together in the 3D structure of the protein [11,21] or to understand the impact on the protein function (see discussion). Using the crystal structure of the ACTB monomer, we mapped the amino acid alterations in ACTB and ACTG1 resulting from missense mutations observed in the DLBCL and multiple myeloma patient samples (Figure 4a,b). Most of the beta-cytoplasmic actin mutations associated with DLBCL are in subdomain (SD) 1 (subdomains as defined in [22] and indicated in Figure 4a–c, together with important regions indicated in Figure 4c,d). There are fewer mutations in SD2 and SD3 and only one in SD4. The DLBCL mutations in ACTG1 map mostly to SD1 and SD2 whereas these in multiple myeloma are almost exclusively in SD1. Thus, mutations in the cytoplasmic actins in DLBCL and multiple myeloma samples are not distributed evenly across actin’s primary and tertiary structures.

## 3. Discussion

Using patient data available at cBioPortal we show that mutations in *ACTB* and *ACTG1* in hematological cancers all occur in lymphoid cancers. *ACTB* mutations are found primarily in DLBCL and *ACTG1* mutations are most frequent in multiple myeloma. For beta-cytoplasmic actin the DLBCL mutations occur most often in subdomain (SD) 1, followed by their presence in SD2 and SD3, and they hardly occur in SD4. For gamma-cytoplasmic actin in DLBCL, mutations mainly occur in SD1 and SD2 and for multiple myeloma almost exclusively in SD1. Below we discuss the potential impact of these mutations on the functioning of actin.

### 3.1. Structural Interpretation of the Mutational Profile and Possible Impact on Functional Properties of Actin

From Figure 4a,b it is evident that the DLBCL or multiple myeloma mutations in ACTB or ACTG1 are not randomly distributed over the 3D-actin structure. Research on these individual mutant actins will be necessary to understand the functional consequences of the mutations and the potential roles in disease progression. Indeed, actin has a complex biochemistry [1]. It has a complex folding pathway [23], an intricate ATP-dependent polymerization cycle and it interacts with numerous proteins [1,24,25]. For the discussion below we assume that the mutated cytoplasmic actins are properly folded. Indeed, despite an evolutionary very conserved structure [2] the beta-cytoplasmic actin protein molecule displays a remarkable structural tolerance towards introduced mutations [26].

A number of F-actin filament structures have been published [4,5,6,27,28,29]. These F-actin structures largely agree on the regions that establish the polymer contacts although they differ in detail (in part because of different resolutions, in part by different experimental conditions: e.g., different ions, nucleotides, presence or absence of actin binding proteins/drugs). Several regions of the primary structure have been found to be important for filament formation or binding ATP or its hydrolysis [6,27] (Figure 4c, Table 4). For polymer formation these include the D-loop (residues 40–50) in SD2 that undergoes a large conformational change to contact residues in the W-loop (residues 165–172) in SD3 of a neighboring actin subunit in a longitudinal contact (Figure 4d). The H-plug (residues 263–273), which was formerly called the hydrophobic plug, establishes a hydrophilic/electrostatic contact with (positively charged) residues in two other actin subunits across the filament axis: R39 and H40 in SD2 from one subunit and His173 in SD3 from another [5]. With respect to nucleotide binding and hydrolysis and gamma-phosphate release other regions are at play. The P-loop 1 (13–16) and P-loop 2 (156–159) are important for amongst other positioning the beta-phosphate. The hinge regions, which consists of the hinge loop (residues 335–337) and the hinge helix (residues 137–145) are important for flattening of actin subunits upon polymer formation. The hinge loop also contains K336 which contacts the adenosine base, and the hinge helix contains Q137 which is considered as the catalytic residue involved in the hydrolysis reaction [6] leading to gamma-phosphate release via the exit tunnel [27]. This release is regulated by the sensor loop (residues 71–77) containing the methylated His73 and by R177 at the end of the exit tunnel. The coupling of conformational changes of the W- and D-loops with the status of the nucleotide are communicated via amongst other the Pro-rich region (residues 108–112) in SD1.

Upon considering the beta-cytoplasmic actin mutations reported in DLBCL patient samples, more than one third (11 out of 27) occur in regions or sites that are important in filament formation or ATP-binding and hydrolysis or phosphate release (Figure 4a,b, Table 4). This excludes residues that are immediately adjacent to these amino acids or regions and of which it can be expected they affect the local conformation in the substructures mediating the contacts. For instance, four mutations are found in the D-loop and two other mutations: D51A and L65F are spatially adjacent to this loop. The latter amino acid also is immediately C-terminal from I64 that stabilizes the hydrophobic contact with SD3. Conversely, T148 in SD3 makes a contact with D-loop residue M44 and is mutated to alanine in one patient sample. Also, the mutation of A135 to V can affect polymer formation as it is close to I136 and V139. Both are part of the hydrophobic interaction with the D-loop in the structure of the ADP-filament [6]. This mutation and these of M132 and Y133 are situated in the beta-strand leading to the hinge helix and thus can also have impact on the latter’s rotation required for the structural alterations of the actin subunits upon (de)polymerization [4,5,6]. Alternatively, mutation of these residues and in particular of Y133 (to N) can disrupt the π-SH interaction of this aromatic residue with the side chain of C374 observed in the ADP-Pi-filament. The latter residue switches to an interaction with the D-loop in the ADP-filament [6].

With respect to nucleotide binding, mutation of G13 and G15 in the P1-loop and of G156 in the P2-loop to more bulky amino acids can affect the local structures of both hairpins. These have additional mutations (G20A/D and G150S) more distal from the contact with the β-phosphate but at present it is hard to predict whether these mutations affect nucleotide binding or another aspect of the ATP-cycle in actin. In the N-terminal hairpin structure G20 is spatially close to D11 for which it has been experimentally shown that its mutation increases nucleotide release [30]. The hinge loop (335–337), involved in conformational switches between the G- and F-actin conformation, and its surroundings appear a small hotspot with three mutations in beta-cytoplasmic actin. However, K336 which contacts the adenine base is not mutated. Thus, the majority of the DLBCL mutations can potentially structurally affect polymer formation or influence nucleotide binding or hydrolysis dynamics. It has to be mentioned that from our current knowledge of actin it is difficult to predict whether these mutations will influence polymer formation or nucleotide turnover in a positive or negative manner.

The picture for γ-cytoplasmic actin is somewhat different. Similar to the ACTB mutations in DLBCL, a part of the ACTG1 mutations can affect a polymer contact, especially the ones near the D-loop (I64N, R62G, R62C, L65M) or in the Pro-rich region (L110V, P112S) (Figure 4b, Table 4). The gamma-cytoplasmic mutations in multiple myeloma, however, almost exclusively map to SD1 and mainly in the N-terminal sequence (Figure 4b). Also, the two beta-cytoplasmic actin multiple myeloma mutations are located at the extreme N-terminus (Figure 4a). This region is essential for binding to myosins via electrostatic interactions [31,32,33,34]. Thus, loss of charge (E3A) or a charge reversal mutation (E4K) in the N-terminus of ACTG1 is likely to influence actomyosin binding. Cytoplasmic actins can interact with multiple myosin types in many cellular processes like cargo movement, cell migration, adhesion, morphogenesis and cytokinesis [35]. However, at present it is hard to pinpoint which actomyosin interaction in myeloma cells would be affected and whether this plays a role in disease progression. Thus, most cytoplasmic actin mutations found in multiple myeloma might influence myosin binding (or possibly other actin-binding proteins contacting the N-terminus), whereas mutations found in DLBCL are more likely to affect actin polymer formation or stability.

### 3.2. A Comparison with ACTB and ACTG1 Mutations in Developmental Diseases

Mutations in actins have previously not been mapped across different cancers or within one cancer (sub)type, however, mutations in cytoplasmic actins have been linked to different developmental diseases such as deafness-dystonia [36], progressive deafness [36,37,38,39], intellectual disability due to haploinsufficiency [7], Becker’s nevus syndrome due to low-grade mosaic postzygotic ACTB hotspot mutations [40], Baraitser-Winter cerebrofrontofacial syndrome (BWCFF) due to mutations in exons 2–4 [9,41] and ACTB-associated thrombocytopenia (ACTB-AST) due to mutations in exon 5 and 6 [42]. These are all rare diseases with very few diagnosed patients having mostly heterozygous missense mutations in *ACTB* or *ACTG1.* Only two sites of the DLBCL associated mutations overlap exactly with mutations in BWCFF (L65 in ACTB and ACTG1) or ACTB-AST (S338). Similar to DLBCL or multiple myeloma patients, BWCFF cases carry mutations in SD1. Yet, in BWCFF mutations in SD4 are more frequent and only one occurs in SD3, whereas for DLBCL this is reversed. Likewise, all known ACTB-AST associated mutations are located in the extreme C-terminus of actin (at the base of SD1 in Figure 4c) and this is shared with only a minor amount of the DLCBL associated mutations in beta-cytoplasmic actin and not a single one in gamma-cytoplasmic actin. This indicates that these diseases each have a distinct mutational pattern.

For only a few of the congenital mutations functional and/or biochemical consequences have been studied and, interestingly, some of these map close to the positions of the mutations in ACTB or ACTG1 in DLBCL or multiple myeloma. Most of these studies are consistent with the production of functional actin mutants that alter F-actin dynamics. Cells from lymphoblastoid cell lines from two BWCFF patients carrying the R196H mutation in ACTB or the S155F mutation in ACTG1, have a higher F-actin content, more F-actin rich filopodia-like protrusions and altered sensitivity to treatment with the actin depolymerizing drug latrunculin A (decreased in R196H, increased in S155F) compared to control cells, suggesting an effect on actin polymerization kinetics [9]. At low density, dermal fibroblasts from ACTB-AST patients carrying mutation A331V_fs*27 or p.S338_I341del are smaller than control cells consistent with cytoskeletal defects in establishing cell morphology. They also show reduced migratory capacity. Platelets carrying these mutations are enlarged and show increased recruitment of the actin binding proteins non-muscle myosin 2A, filamin A and alpha-actinin 1 [42]. Patient lymphoblasts carrying the R183W mutation demonstrate long tapering processes and treatment with Latrunculin A is less effective than in control cells [36]. Actins carrying this mutation are impaired in forming long stable filaments [43]. Lymphocytes from a patient carrying an ACTB E117K mutation seen in an atypical form of Baraitser-Winter syndrome, show decreased ability to adhere to fibronectin surfaces and show less protrusive structures. Biochemical experiments show that this actin is almost completely resistant to latrunculin A treatment and demonstrates faster polymerization, indicating increased filament stability [44]. Also the eight ACTG1 mutations (T89I, K118N, K118M, E241K, P264L, T278I, P332A, V370A) involved in progressive hearing loss in autosomal dominant isolated neurosensory deafness type DFNA lead to specific changes in polymerization and F-actin severing by the actin-binding protein cofilin [39,45]. These examples from congenital diseases in which cytoplasmic actin mutations affect actin polymerization and/or interactions with ABPs, thereby altering cell morphology and/or cell migration capacity, reinforce the possibility that the ACTB and ACTG1 mutations in DLBCL and multiple myeloma can have a functional contribution to the progression of these lymphoid cancers.

### 3.3. ACTB and ACTG1 Mutations: More Than Passenger Mutations in DLBCL and Multiple Myeloma?

ACTB is deregulated in multiple cancers and the resulting alterations to the cytoskeleton caused by altered expression and polymerization of ACTB have been proposed to be associated with the invasiveness and metastasis of cancers [10]. This is also based on seminal research in the 1980’s in which a G245D mutation was discovered in ACTB [46] and found to be capable of converting immortal human fibroblasts into stably tumorigenic cells with increased tropomyosin expression, which is characteristic of the neoplastic phenotype [47]. Independently, the extremely rare actinopathy in Baraitser-Winter syndrome has been proposed as a cancer-predisposing disorder, especially for hematological malignancies. This is based on three patients that developed leukemia or lymphoma, although the small sample size needs to be taken into account [41,48]. It was suggested by [41] that ACTB has at most a marginal role in sporadic hematologic carcinogenesis because a screening of 95 B-cell acute lymphoblastic leukemia (B-ALL) samples identified no *ACTB* mutations. cBioPortal data indeed indicate a single mutation in B-ALL, however, we show that the *ACTB* mutations found in lymphoid cancers almost exclusively occur in mature B-cell neoplasms (Figure 3a). Since B-ALL arises from pregerminal B-cells (immature cells) and DLBCL from post-follicle center B-cells (mature cells), this is compatible with the suggestion that dysregulated actin dynamics during maturation of B-cells could lead to B-cell malignancy. During maturation B-cells are highly motile and this motility is dependent on actin polymerization in function of changing cell shape and coordination of migration [49]. This is also in line with our observation that *ACTB* is as frequently mutated as a recognized driver in DLCBL: *RHOA* [16,17]. Interestingly, ACTB is downstream of RHOA signaling and also RHOA is important in cell migration [50]. In addition, *ACTB* and *RHOA* mutations are categorized in the same subtype of DLBCL identified by [17], and *ACTB* and *RHOA* were found to be driver genes in DLBCL but *ACTB* was not elaborated on by [16]. Therefore, *ACTB* mutations can play a role in DLBCL progression. Of note is that, in a multiple myeloma study, *ACTG1* met the criteria for being a driver in this disease although this was not explicitly mentioned by the authors [13]). In a later study, *ACTG1* was also recognized as a potential driver in multiple myeloma [15]. Together, this suggests that *ACTB* and *ACTG1* mutations are potentially more than passenger mutations in DLBCL and multiple myeloma, respectively. However, experiments are needed to show this causative effect and to rule out the possibility that the observed enrichment of *ACTB* or *ACTG1* mutations in these two cancer types reflect a higher tolerance for such mutations compared to other cancer types in which they are therefore less detected. This or the causality of the observed mutations in DLBCL and multiple myeloma need further investigation by future screening studies of additional patients and functional studies of ACTB or ACTG1 mutants in appropriate models or patient material.

## 4. Materials and Methods

### 4.1. Queries in cBioPortal

All used data from cBioPortal (http://www.cbioportal.org/index.do) [51,52] were retrieved on 26 January 2020. Part of these data were generated by the TCGA Research Network: https://www.cancer.gov/tcga. Several queries were used in which the selected cancer studies, the selected molecular profiles (mutations or copy number alterations (CNA)) and the entered genes varied. The selected patient/case set was invariably set to ‘all’. For the selection of studies, the box ‘Curated set of non-redundant studies’ was checked (except for one query in Table S3). Other selections made in the queries are indicated in the text, table headings or figure captions.

The 174 cancer studies, sometimes referred to in the main text as ‘all studies’, excludes the non-redundant PanCancer studies MSK-IMPACT Clinical Sequencing Cohort (MSKCC, [53]) in which actin genes were not profiled and the Pediatric Pan-cancer study (Columbia U, [54]) because it contains a limited number of samples (103) most often of a single cancer type. The analyzed set of 174 studies contained 33,923 patients/35,649 samples in total. Note that only few studies or patient samples contain both mutational profiles and CNA profiles, therefore the ‘number of patients profiled’ can be lower than the total number of patients.

Selection of molecular profiles: when specifically referring to mutations, the checkbox ‘Mutation’ was selected in the query. When specifically referring to copy number alterations, the checkbox ‘Copy number alterations’ was selected in the query.

### 4.2. Handling of Query Results from cBioPortal

The results’ output from each query in cBioPortal is divided into several tabs of which we used the following: Oncoprint, Cancer Types Summary, and Mutations. Each table heading or figure caption indicates from which tab the presented data originates. Oncoprint figures and lollipop figures were downloaded directly from the Oncoprint tab and Mutations tab, respectively. The percentages in the Oncoprints always refer to ‘events per patient’. The tab ‘Cancer Types Summary’ has three distinct data analysis possibilities: ‘cancer study’, ‘cancer type’ and ‘cancer type detailed’ for the genes selected in the query, taken either separately or combined. In addition, there is a possibility to switch the Y-axis between frequency (%) or absolute counts of cases. Each of these options is informative and used in this paper and the type of data is always explicitly mentioned. The X-axis was always set to ‘sort by Y-axis values’, the minimum number of total cases was always set to 10 and the minimum percent of altered cases was always set to 0.

### 4.3. D Structures

The Protein Data Bank (PDB) (https://www.rcsb.org/structure/) was used to retrieve the 3D-structures of the actin monomer (PDB: 2BTF, actin-profilin complex) [55] and the actin polymer (PDB: 6djm) [6]. These were further processed with Viewerlite 5.0 (Accelrys, San Diego, California, USA). In 2BTF, the profilin molecule was removed.

## 5. Conclusions

Our results show that mutations in the cytoplasmic actins in hematological cancers occur specifically in lymphoid cancers and not in myeloid cancers, implying these mutations need dedicated studies in the field of lymphoma research. Compared to other lymphoid cancer types, mutations in *ACTB* and *ACTG1* are most frequent in DLBCL and multiple myeloma, respectively. The structural mapping suggests that the mutations are in regions that affect different actin properties (e.g., polymer formation or binding myosin) that potentially alter cell behavior (e.g., morphology, adhesion, proliferation or migration). At present it is unclear whether the mutations are causative of DLCBL/multiple myeloma or are involved in the disease progression but we propose that future research on these diseases takes into account the role of the actin cytoskeleton, especially the RHOA-signaling axis and *ACTB* mutations in DLCBL patients, and *ACTG1* mutations in multiple myeloma patients.

## Figures and Tables

**Table 1:** Frequency (altered/profiled patients, in %) of alterations in actin genes in the 174 selected cancer studies (Table S1) in cBioPortal. CNA = copy number alterations, Mut = somatic mutations. Data derived from the Oncoprint tab.

	CNA + Mut	CNA	Mut
Number of patients profiled (100%)	29,522	18,166	24,471
*ACTB*	2.2	2	1.2
*ACTG1*	2	2.4	0.7
*ACTA2*	1.4	1.6	0.5
*ACTG2*	0.8	0.5	0.7
*ACTA1*	4	5	0.7
*ACTC1*	1.3	0.8	0.9

**Table 2:** Frequency of cases (altered/profiled patients, in %) displaying somatic mutations in *ACTB* and/or *ACTG1* per study including the rank (out of 174) of these studies. For clarity only the top-ranking cancer studies are given; complete information is available at cBioportal. NR = not ranked. For full study names see Table S1. Data derived from the Cancer Types Summary tab.

Study	*ACTB* + *ACTG1*		*ACTB*		*ACTG1*		cBioPortal Division
	%	Rank	%	Rank	%	Rank	
BCC (UNIGE 2016)	22.22	1	15.08	1	7.94	2	Basal cell carcinoma
DLBCL (TCGA PanCan)	17.07	2	9.76	4	9.76	1	Diffuse Large B-Cell Lymphoma
DLBCLNOS (DFCI)	13.33	3	10.37	2	2.22	15	Diffuse Large B-Cell Lymphoma
cSCC (MD ANDERSON 2014)	10.26	4	5.13	8	7.69	3	Cutaneous Squamous Cell Carcinoma
Acyc (MGH 2016)	10.00	5	10.00	3	0.00	NR	Adenoid Cystic Carcinoma (small dataset)
Bladder (BGI 2013)	9.09	6	7.07	5	2.02	16	Bladder Urothelial Carcinoma
BLCA (Cornell 2016)	8.33	7	2.78	20	5.56	4	Urothelial Carcinoma
COAD (CPTAC-2 2019)	5.66	12	1.89	32	3.77	5	Colon Cancer

**Table 3:** Frequency (altered/profiled patients, in %) of somatic mutations of the indicated genes in the selected 174 studies (all studies) (Table S1), the lymphoid (L) and myeloid (M) studies (Table S2), and DLBCL cases. Data derived from the OncoPrint tabs except for these in column ‘DLBCL’ which were derived as in Figure 3c.

	All Studies	L + M	M	L	DLBCL (Absolute Counts)
Number of patients profiled (100%)	29,473	4,179	1,134	3045	1250
*ACTB*	1.2	1	0	1.3	2.7 (34)
*ACTG1*	0.7	0.4	0	0.6	0.6 (8)
*RHOA*	0.9	0.9	0	1.3	3.1 (39)
*RHOB*	0.4	0	0	0	0.1 (1)
*RHOC*	0.2	0	0	0.1	0.1 (1)
*RAC1*	0.6	0.1	0	0.1	0.1 (1)
*RAC2*	0.4	0.2	0.2	0.2	0.3 (4)
*RAC3*	0.2	0.1	0.2	0	0.1 (1)
*CDC42*	0.3	0.1	0.1	0.1	0.2 (2)

**Table 4:** Overview of cytoplasmic actin mutations in DLBCL (regular type) and multiple myeloma (bold) in regions involved in polymer formation, binding of ATP or phosphate release. – indicates no mutation is present.

	Actin Region or Subdomain (SD) Involved	Mutation in ACTB	Mutation in ACTG1
Polymer contact	SD2:D-loop (40–50)	M44T, M44I, G46D, M47L	-
	Other SD2 contacts with SD3	K61N	I64N, R62G
	SD3: W-loop (165–172)	-	-
	Other SD3 contacts with SD2	T148A	-
	Pro-rich loop (108–112)	-	L110V, P112S
	SD4 H-plug (263–273)	-	-
	SD2 H-plug contact	-	**R39I**
	SD1–3: Hinge Helix (137–145)	-	*-*
	SD3-1: Hinge Loop (P335-S337)	Y337S	*-*
ATP-binding, phosphate release	P-loop1 (13–16)	G13A, G15D	-
	P-loop2 (156–159)	G156S	-
	SD3-1: Hinge Loop with K336 contacting the adenosine base	Y337S	-
	Sensor loop (71–77)	-	-

## Supplementary Materials

Supplementary Materials can be found at https://www.mdpi.com/1422-0067/21/9/3093/s1. Table S1. List of the 174 studies selected in cBioPortal. Table S2. Lymphoid and myeloid cancer studies in cBioPortal. Table S3. Summary of ACTB and ACTG1 mutations found in DLBCL and multiple myeloma patients. Figure S1. Oncoprint of the six human actin genes in the selected 174 non-redundant studies available at cBioportal. Figure S2. Oncoprint of *ACTB*, *ACTG1*, *RHOA* and *RAC1* in the 4 DLBCL studies at cBioportal. Figure S3. Lollipop views of actin mutations.

## Abbreviations

ACTB-AST	ACTB-associated thrombocytopenia
B-ALL	B-cell acute lymphoblastic leukemia
BWCFF	Baraitser-Winter Cerebrofrontofacial Syndrome
CNA	Copy number alteration
DLBCL	Diffuse large B-cell lymphoma
F-actin	Filamentous actin
PDB	Protein Data Bank
SD	Subdomain
