# Supplementary material for: Prevalence of Cytoplasmic Actin Mutations in Diffuse Large B-Cell Lymphoma and Multiple Myeloma: A Functional Assessment Based on Actin Three-Dimensional Structures
Source: Int J Mol Sci. 2020 Apr 27;21(9):3093. doi: 10.3390/ijms21093093 (PMC7247664; doi:10.3390/ijms21093093)
Supplement: Supplementary file 1 [file ijms-21-03093-s001.pdf]

Supplementary Material

Table S1. List of the 174 studies selected in cBioPortal.

	Cancer study	Shown in Table	Shwon in Figure
Adrenal Gland	Adenoid Cystic Carcinoma Project (2019)		
	Adrenocortical Carcinoma (TCGA, PanCancer Atlas)		
Ampulla of Vater	Ampullary Carcinoma (Baylor College of Medicine, Cell Reports 2016)		
Biliary Tract	Cholangiocarcinoma (National Cancer Centre of Singapore, Nat Genet 2013)		
	Cholangiocarcinoma (National University of Singapore, Nat Genet 2012)		
	Cholangiocarcinoma (TCGA, PanCancer Atlas)		
	Intrahepatic Cholangiocarcinoma (JHU, Nat Genet 2013)		
	Intrahepatic Cholangiocarcinoma (Shanghai, Nat Commun 2014)		
	Gallbladder Carcinoma (Shanghai, Nat Genet 2014)		
Bladder/Urinary Tract	Bladder Cancer (MSKCC, Eur Urol 2014)		
	Bladder Cancer (MSKCC, J Clin Onco 2013)		
	Bladder Urothelial Carcinoma (BGI, Nat Genet 2013)	x	x
	Bladder Urothelial Carcinoma (DFCI/MSKCC, Cancer Discov 2014)		
	Bladder Urothelial Carcinoma (TCGA, PanCancer Atlas)		x
	Urothelial Carcinoma (Cornell/Trento, Nat Gen 2016)	x	x
	Upper Tract Urothelial Cancer (MSK, Eur Urol 2015)		
	Upper Tract Urothelial Carcinoma (Cornell/Baylor/MDACC, Nat Comm 2019)		
Bone	Ewing Sarcoma (Institut Curie, Cancer Discov 2014)		
	Pediatric Ewing Sarcoma (DFCI, Cancer Discov 2014)		
Bowel	Colorectal Adenocarcinoma (DFCI, Cell Reports 2016)		
	Colorectal Adenocarcinoma (Genentech, Nature 2012)		
	Colorectal Adenocarcinoma (TCGA, PanCancer Atlas)		
	Colorectal Adenocarcinoma Triplets (MSKCC, Genome Biol 2014)		
	Colon Adenocarcinoma (CaseCCC, PNAS 2015)		
	Colon Cancer (CPTAC-2 Prospective, Cell 2019)	x	
Breast	Breast Fibroepithelial Tumors (Duke-NUS, Nat Genet 2015)		
	Breast Cancer (METABRIC, Nature 2012 & Nat Commun 2016)		
	Breast Cancer (MSKCC, 2019)		
	Breast Invasive Carcinoma (British Columbia, Nature 2012)		
	Breast Invasive Carcinoma (Broad, Nature 2012)		
	Breast Invasive Carcinoma (Sanger, Nature 2012)		
	Breast Invasive Carcinoma (TCGA, PanCancer Atlas)		
	Metastatic Breast Cancer (INSERM, PLoS Med 2016)		

	The Metastatic Breast Cancer Project (Provisional, October 2018)		
	Adenoid Cystic Carcinoma of the Breast (MSKCC, J Pathol. 2015)		
CNS/Brain	Brain Lower Grade Glioma (TCGA, PanCancer Atlas)		
	Glioma (MSK, 2018)		
	Low-Grade Gliomas (UCSF, Science 2014)		
	Glioblastoma Multiforme (TCGA, PanCancer Atlas)		
	Medulloblastoma (Broad, Nature 2012)		
	Medulloblastoma (PCGP, Nature 2012)		
	Medulloblastoma (Sickkids, Nature 2016)		
Cervix	Cervical Squamous Cell Carcinoma (TCGA, PanCancer Atlas)		
Esophagus/Stomach	Esophageal Squamous Cell Carcinoma (ICGC, Nature 2014)		
	Esophageal Squamous Cell Carcinoma (UCLA, Nat Genet 2014)		
	Gastric Adenocarcinoma (TMUCIH, PNAS 2015)		
	Esophageal Adenocarcinoma (DFCI, Nat Genet 2013)		
	Esophageal Adenocarcinoma (TCGA, PanCancer Atlas)		
	Stomach Adenocarcinoma (Pfizer and UHK, Nat Genet 2014)		
	Stomach Adenocarcinoma (TCGA, PanCancer Atlas)		
	Stomach Adenocarcinoma (U Tokyo, Nat Genet 2014)		
Eye	Uveal Melanoma (QIMR, Oncotarget 2016)		
	Uveal Melanoma (TCGA, PanCancer Atlas)		
Head and Neck	Head and Neck Squamous Cell Carcinoma (Broad, Science 2011)		
	Head and Neck Squamous Cell Carcinoma (Johns Hopkins, Science 2011)		
	Head and Neck Squamous Cell Carcinoma (TCGA, PanCancer Atlas)		
	Oral Squamous Cell Carcinoma (MD Anderson, Cancer Discov 2013)		
	Nasopharyngeal Carcinoma (Singapore, Nat Genet 2014)		
	Adenoid Cystic Carcinoma (FMI, Am J Surg Pathl. 2014)		
	Adenoid Cystic Carcinoma (JHU, Cancer Prev Res 2016)		
	Adenoid Cystic Carcinoma (MDA, Clin Cancer Res 2015)		
	Adenoid Cystic Carcinoma (MGH, Nat Gen 2016)	x	x
	Adenoid Cystic Carcinoma (MSKCC, Nat Genet 2013)		
	Adenoid Cystic Carcinoma (Sanger/MDA, JCI 2013)		
Kidney	Clear Cell Renal Cell Carcinoma (DFCI, Science 2019)		
	Kidney Renal Clear Cell Carcinoma (BGI, Nat Genet 2012)		
	Kidney Renal Clear Cell Carcinoma (IRC, Nat Genet 2014)		
	Kidney Renal Clear Cell Carcinoma (TCGA, PanCancer Atlas)		
	Renal Clear Cell Carcinoma (UTokyo, Nat Genet 2013)		
	Kidney Chromophobe (TCGA, PanCancer Atlas)		
	Kidney Renal Papillary Cell Carcinoma (TCGA, PanCancer Atlas)		
	Renal Non-Clear Cell Carcinoma (Genentech, Nat Genet 2014)		
	Unclassified Renal Cell Carcinoma (MSK, Nature 2016)		
	Pediatric Rhabdoid Tumor (TARGET, 2018)		
	Rhabdoid Cancer (BCGSC, Cancer Cell 2016)		
	Pediatric Wilms' Tumor (TARGET, 2018)		
Liver	Hepatocellular Adenoma (INSERM, Cancer Cell 2014)		
	Hepatocellular Carcinomas (INSERM, Nat Genet 2015)		

	Liver Hepatocellular Adenoma and Carcinomas (MSK, PLOS One 2018)		
	Liver Hepatocellular Carcinoma (AMC, Hepatology 2014)		
	Liver Hepatocellular Carcinoma (RIKEN, Nat Genet 2012)		
	Liver Hepatocellular Carcinoma (TCGA, PanCancer Atlas)		
Lung	Thoracic PDX (MSK, Provisional)		
	Small Cell Lung Cancer (Johns Hopkins, Nat Genet 2012)		
	Small Cell Lung Cancer (U Cologne, Nature 2015)		
	Small-Cell Lung Cancer (Multi-Institute, Cancer Cell 2017)		
	Non-Small Cell Lung Cancer (MSK, Cancer Cell 2018)		
	Non-Small Cell Lung Cancer (TRACERx, NEJM 2017)		
	Non-Small Cell Lung Cancer (University of Turin, Lung Cancer 2017)		
	Lung Adenocarcinoma (Broad, Cell 2012)		
	Lung Adenocarcinoma (TCGA, PanCancer Atlas)		
	Lung Adenocarcinoma (TSP, Nature 2008)		
	Lung Squamous Cell Carcinoma (TCGA, PanCancer Atlas)		
Lymphoid	Acute Lymphoblastic Leukemia (St Jude, Nat Genet 2016)		
	Pediatric Acute Lymphoid Leukemia - Phase II (TARGET, 2018)		
	Chronic Lymphocytic Leukemia (Broad, Cell 2013)		
	Chronic Lymphocytic Leukemia (Broad, Nature 2015)		
	Chronic Lymphocytic Leukemia (IUOPA, Nature 2015)		
	Chronic lymphocytic leukemia (ICGC, Nature Genetics 2011)		
	Cutaneous T Cell Lymphoma (Columbia U, Nat Genet 2015)		
	Diffuse Large B cell Lymphoma (DFCI, Nat Med 2018)	x	x
	Diffuse Large B-Cell Lymphoma (Duke, Cell 2017)		
	Diffuse Large B-Cell Lymphoma (TCGA, PanCancer Atlas)	x	x
	Diffuse Large B-cell Lymphoma (BCGSC, Blood 2013)		
	Mantle Cell Lymphoma (IDIBIPS, PNAS 2013)		
	Multiple Myeloma (Broad, Cancer Cell 2014)		
	Non-Hodgkin Lymphoma (BCGSC, Nature 2011)		
	Primary Central Nervous System Lymphoma (Mayo Clinic, Clin Cancer Res 2015)		
Myeloid	Acute myeloid leukemia or myelodysplastic syndromes (WashU, 2016)		
	Acute Myeloid Leukemia (OHSU, Nature 2018)		
	Acute Myeloid Leukemia (TCGA, PanCancer Atlas)		
	Pediatric Acute Myeloid Leukemia (TARGET, 2018)		
	Histiocytosis Cobimetinib (MSK, Nature 2019)		
	Myelodysplasia (UTokyo, Nature 2011)		
	Myeloproliferative Neoplasms (CIMR, NEJM 2013)		
Other	MSS Mixed Solid Tumors (Broad/Dana-Farber, Nat Genet 2018)		
	Metastatic Solid Cancers (UMich, Nature 2017)		
	Pediatric Pan-Cancer (DKFZ, Nature 2017)		
	Pediatric Preclinical Testing Consortium (Maris, 2019)		
	SUMMIT - Neratinib Basket Study (Multi-Institute, Nature 2018)		
Ovary/Fallopian Tube	Ovarian Serous Cystadenocarcinoma (TCGA, PanCancer Atlas)		
	Small Cell Carcinoma of the Ovary (MSKCC, Nat Genet 2014)		

Pancreas	Acinar Cell Carcinoma of the Pancreas (JHU, J Pathol 2014)		
	Cystic Tumor of the Pancreas (Johns Hopkins, PNAS 2011)		
	Pancreatic Adenocarcinoma (QCMG, Nature 2016)		
	Pancreatic Adenocarcinoma (TCGA, PanCancer Atlas)		
	Pancreatic Cancer (UTSW, Nat Commun 2015)		
	Insulinoma (Shanghai, Nat Commun 2013)		
	Pancreatic Neuroendocrine Tumors (Johns Hopkins University, Science 2011)		
	Pancreatic Neuroendocrine Tumors (Multi-Institute, Nature 2017)		
Peripheral Nervous System	Malignant Peripheral Nerve Sheath Tumor (MSKCC, Nat Genet 2014)		
	Neuroblastoma (AMC Amsterdam, Nature 2012)		
	Neuroblastoma (Broad, Nature 2015)		
	Pediatric Neuroblastoma (TARGET, 2018)		
Pleura	Mesothelioma (TCGA, PanCancer Atlas)		
	Pleural Mesothelioma (NYU, Cancer Res 2015)		
Prostate	Prostate Cancer (MSK, 2019)		
	Metastatic Prostate Adenocarcinoma (MCTP, Nature 2012)		
	Metastatic Prostate Adenocarcinoma (SU2C/PCF Dream Team, PNAS 2019)		
	Neuroendocrine Prostate Cancer (Multi-Institute, Nat Med 2016)		
	Prostate Adenocarcinoma (Broad/Cornell, Nat Genet 2012)		
	Prostate Adenocarcinoma (Fred Hutchinson CRC, Nat Med 2016)		
	Prostate Adenocarcinoma (MSKCC, Cancer Cell 2010)		
	Prostate Adenocarcinoma (SMMU, Eur Urol 2017)		
	Prostate Adenocarcinoma (TCGA, PanCancer Atlas)		
	Prostate Adenocarcinoma Organoids (MSKCC, Cell 2014)		
	The Metastatic Prostate Cancer Project (Provisional, December 2018)		
Skin	Basal Cell Carcinoma (UNIGE, Nat Genet 2016)	x	x
	Cutaneous Squamous Cell Carcinoma (DFCI, Clin Cancer Res 2015)		
	Cutaneous Squamous Cell Carcinoma (MD Anderson, Clin Cancer Res 2014)	x	x
	Acral Melanoma (TGEN, Genome Res 2017)		
	Metastatic Melanoma (UCLA, Cell 2016)		
	Melanoma (MSKCC, NEJM 2014)		
	Metastatic Melanoma (DFCI, Science 2015)		
	Metastatic Melanoma (MSKCC, JCO Precis Oncol 2017)		
	Skin Cutaneous Melanoma (Broad, Cell 2012)		
	Skin Cutaneous Melanoma (TCGA, PanCancer Atlas)		
	Skin Cutaneous Melanoma (Yale, Nat Genet 2012)		
	Skin Cutaneous Melanoma (Broad, Cancer Discov 2014)		x
	Desmoplastic Melanoma (Broad Institute, Nat Genet 2015)		
Soft tissue	Pheochromocytoma and Paraganglioma (TCGA, PanCancer Atlas)		
	Sarcoma (MSKCC/Broad, Nat Genet 2010)		
	Sarcoma (TCGA, PanCancer Atlas)		
	The Angiosarcoma Project - Count Me In (Provisional, September 2018)		

	Rhabdomyosarcoma (NIH, Cancer Discov 2014)		
Testis	Testicular Germ Cell Tumors (TCGA, PanCancer Atlas)		
Thymus	Thymic Epithelial Tumors (NCI, Nat Genet 2014)		
	Thymoma (TCGA, PanCancer Atlas)		
Thyroid	Thyroid Carcinoma (TCGA, PanCancer Atlas)		
Uterus	Uterine Corpus Endometrial Carcinoma (TCGA, PanCancer Atlas)		
	Uterine Carcinosarcoma (Johns Hopkins, Nat Commun 2014)		
	Uterine Carcinosarcoma (TCGA, PanCancer Atlas)		
	Uterine Clear Cell Carcinoma (NIH, Cancer 2017)		
Vulva/Vagina	Squamous Cell Carcinoma of the Vulva (CUK, Exp Mol Med 2018)		x

Table S2: Lymphoid and myeloid cancer studies in cBioPortal. The 15 lymphoid and 7 myeloid cancer studies were selected (see Materials and Methods in main text) for the queries in hematological cancers. * indicate studies on DLBCL, ** indicate a study on multiple myeloma.

Studies on	Subtype	Cancer study	Profiled for copy number alterations (# of samples)	Profiled for mutations (# of samples)
Lymphoid cancers	B-lymphoblastic leukemia/ lymphoma	Acute Lymphoblastic Leukemia (St Jude, Nat Genet 2016)		X
		Pediatric Acute Lymphoid Leukemia - Phase II (TARGET, 2018)	X (764)	X (150)
	Non-Hodgkin Lymphoma	Chronic Lymphocytic Leukemia (Broad, Cell 2013)		X
		Chronic Lymphocytic Leukemia (Broad, Nature 2015		X
		Chronic Lymphocytic Leukemia (IUOPA, Nature 2015)		X
		Chronic lymphocytic leukemia (ICGC, Nature Genetics 2011)		X
		Cutaneous T Cell Lymphoma (Columbia U, Nat Genet 2015)		X
		*Diffuse Large B cell Lymphoma (DFCI, Nat Med 2018)		X
		*Diffuse Large B-Cell Lymphoma (Duke, Cell 2017)		X
		*Diffuse Large B-Cell Lymphoma (TCGA, PanCancer Atlas)	X (48)	X (41)
		*Diffuse Large B-cell Lymphoma (BCGSC, Blood 2013) [1]		X
		Mantle Cell Lymphoma (IDIBIPS, PNAS 2013)		X
		**Multiple Myeloma (Broad, Cancer Cell 2014)		X
		Non-Hodgkin Lymphoma (BCGSC, Nature 2011)		X
		Primary Central Nervous System Lymphoma (Mayo Clinic, Clin Cancer Res 2015)		X
Myeloid cancers		Acute myeloid leukemia or myelodysplastic syndromes (WashU, 2016)		X
		Acute Myeloid Leukemia (OHSU, Nature 2018)		X
		Acute Myeloid Leukemia (TCGA, PanCancer Atlas)	X (191)	X (200)
		Pediatric Acute Myeloid Leukemia (TARGET, 2018)	X (240)	X (150)
		Histiocytosis Cobimetinib (MSK, Nature 2019)	X (33)	X (51)
		Myelodysplasia (UTokyo, Nature 2011)		X
		Myeloproliferative Neoplasms (CIMR, NEJM 2013)		X

[1] This study contains 13 cell line samples that contain one ACTB mutation (I151S) and one ACTG1 mutation (R62C). The latter mutation is however also found in one DLBCL patient. The I151 mutation is not taken up in our interpretation of the functional consequences on the 3D-structure in the discussion section of the main text because it is not patient derived.

Table S3. Summary of ACTB and ACTG1 mutations found in DLBCL and multiple myeloma patients. Data are based on four DLBCL studies and one multiple myeloma study indicated with asterisks in Table S2. Mutations in bold were found in two additional DLBCL studies (Diffuse Large B-Cell Lymphoma (Broad, PNAS 2012) and Lymphoid Neoplasm Diffuse Large B-cell Lymphoma (TCGA, Firehose Legacy)) that were not included in all other queries because they are not part of the 'Curated set of non-redundant studies' (see Materials and Methods). Data derived from the mutations tab.

DLBCL		Multiple myeloma	
**ACTB (# of cases)**	**ACTG1**	**ACTB**	**ACTG1 (# of cases)**
D2E	A19V	D3E	E3A
G13A (2)	R62G, **R62C**	I5L	E4_I5delinsDF
G15D	**I64N**		E4K
G20A (2), G20D	**L65M**		L8V
A26V	E93D		F21V
M44T, M44I (4)	P102S		A22P (2)
G46D (3)	**L110V**		D25G
M47L (2)	P112S		R39I
D51A	Q121H		
K61N	X41_splice		
L65F			
D80N			
W86C			
M132T			
Y133N			
A135V			
T148A			
G150S			
G156S			
C217Y			
Y294N			
P333L			
Y337S			
S338P			
K315del			
ACTB-MYC fusion			
X2_splice (2)			

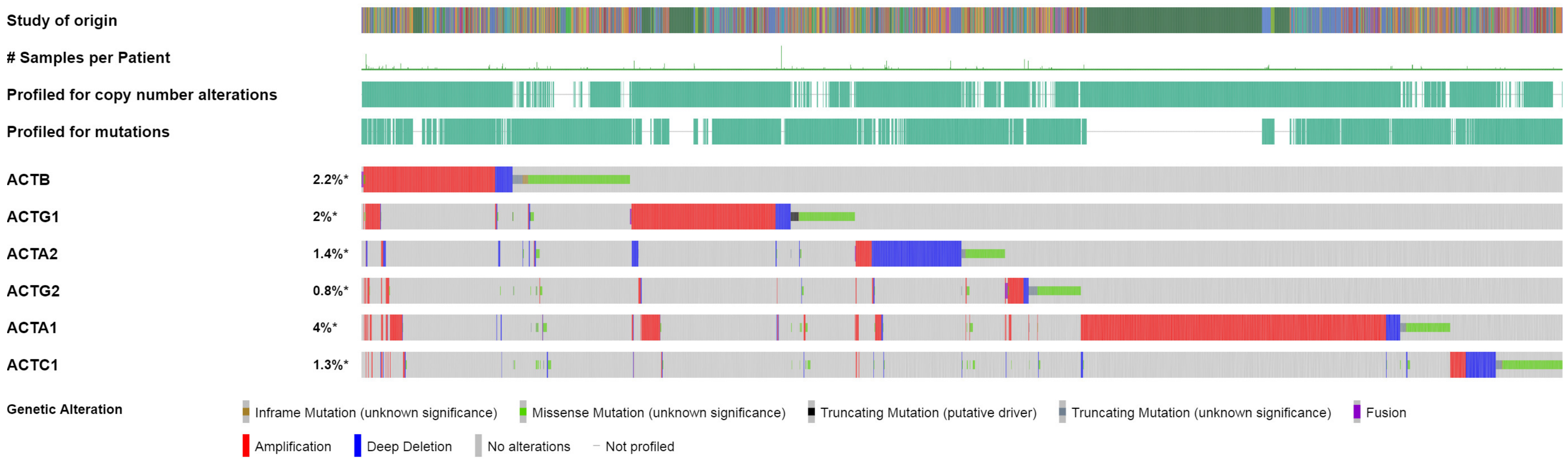

Figure S1. Oncoprint of the six human actin genes in the selected 174 non-redundant studies available at cBioportal. The individual actin genes entered in the query are represented by rows, individual cases (patients) are presented as colums. Only altered cases are shown to reduce the size of the picture. The data were sorted by 'data: mutation type and driver/passenger'. The colors in the top bar represent the different studies of origin included in the query, however, the legend for the colors assigned to the various studies of origin has been omitted to reduce the size of the picture. The height of the columns in the second item from the top is proportional to the number of samples per patient. In the query, both molecular profiles were selected and are shown in the third and fourth item from the top, however, not all patient samples are profiled (white spaces in between the green bars/columns) for both types of genetic alterations: copy number alterations (amplification, deep deletion) or mutations: inframe, missense and truncating mutations or fusions.

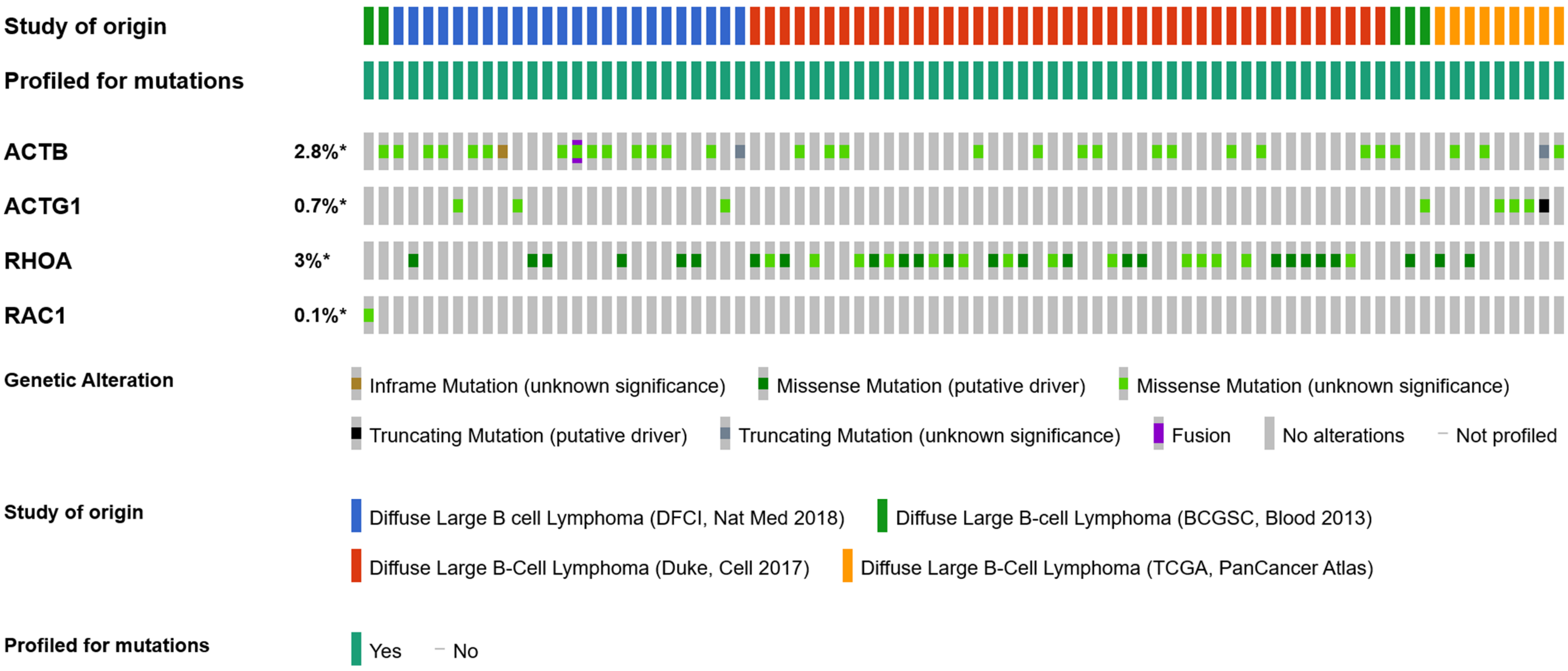

Figure S2. Oncoprint of *ACTB*, *ACTG1*, *RHOA* and *RAC1* in the 4 DLBCL studies at cBioportal. The individual genes entered in the query are represented by rows, individual cases (patients) are presented as colums. Only altered cases are shown. The data were sorted by 'case-id (alphabetical)'. The colors in the top bar represent the different studies of origin included in the query (legend see bottom). In the query, the molecular profile: mutation, was selected. Mutations include inframe, missense and truncating mutations. If only these DLBCL studies are considered the frequency of *ACTB* mutations is 2,8% and that of *ACTG1* 0,7%. For comparison, for *RHOA* and *RAC1* this 3% and 0,1% respectively. These percentages are slightly different from these in Table 3 because for the table additional patient samples were included.

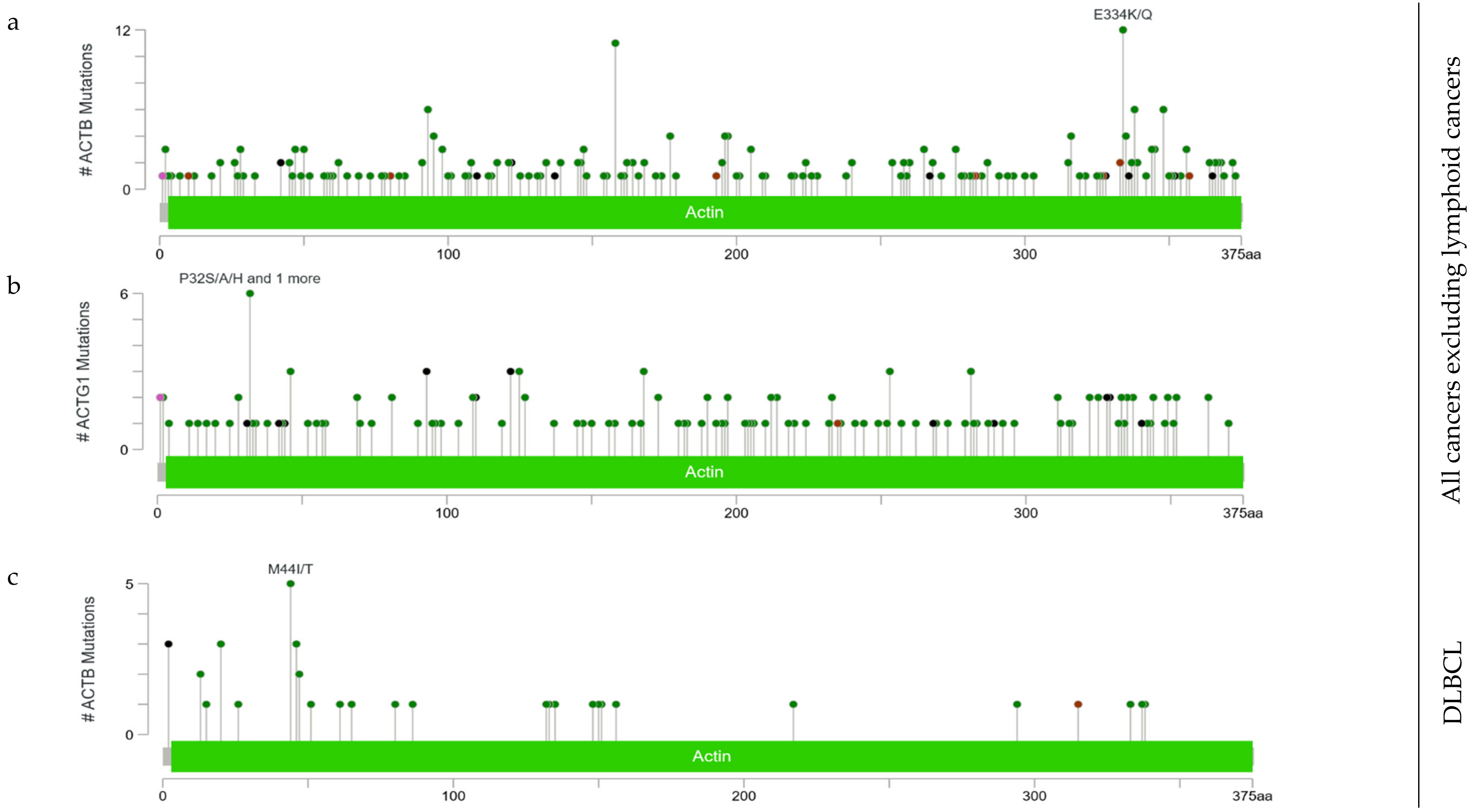
a
E334K/Q
12
0
ACTB Mutations
Actin
0
100
200
300
375aa
b
P32S/A/H and 1 more
6
0
ACTG1 Mutations
Actin
0
100
200
300
375aa
c
M44I/T
5
0
ACTB Mutations
Actin
0
100
200
300
375aa
All cancers excluding lymphoid cancers
DLBCL

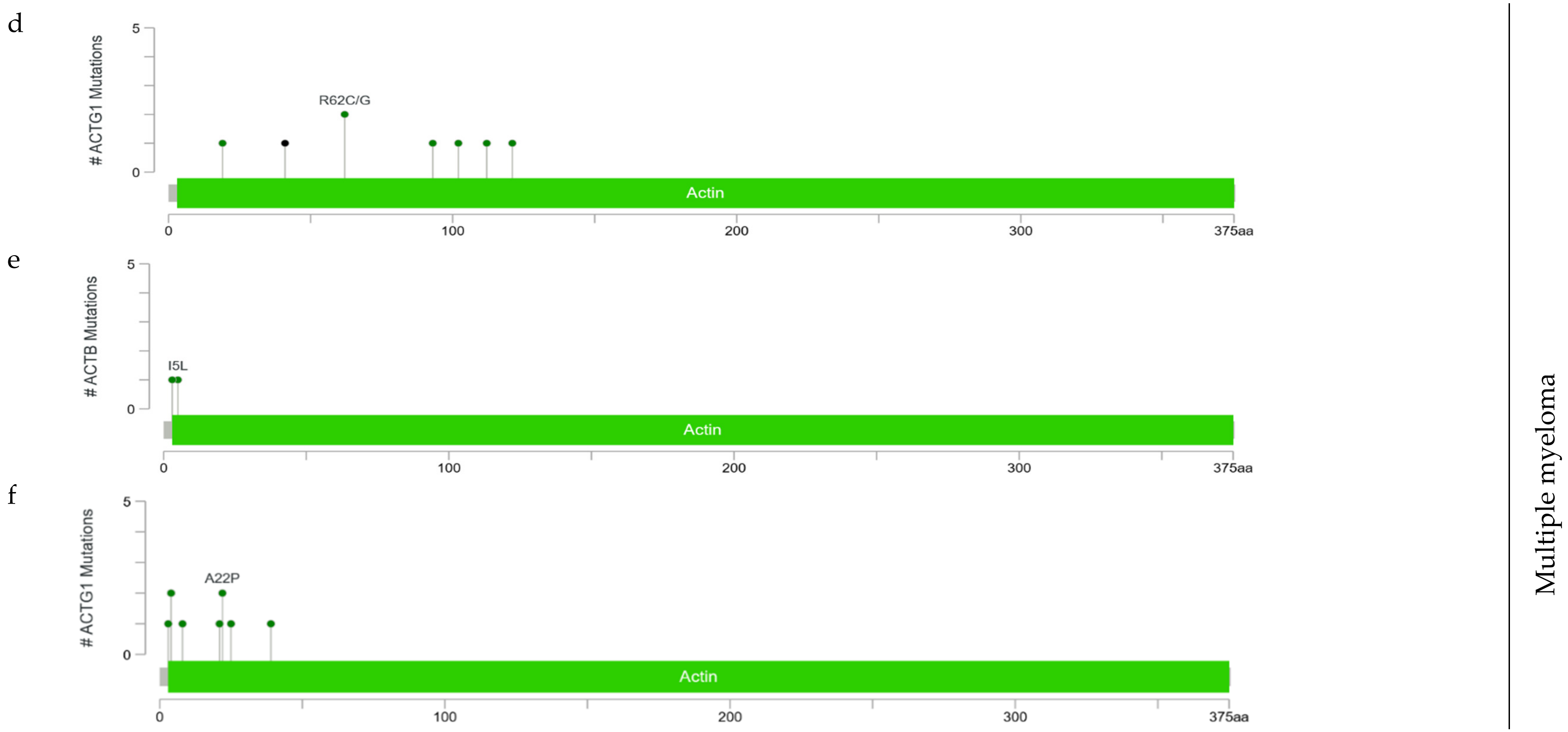

Figure S3. Lollipop views of actin mutations**.** (a, b) Mutations in the selected set of non-redundant studies at cBioPortal (Table S1) excluding mutations from the lymphoid studies (Table S2) in ACTB (a) and ACTG1 (b). Mutations are spread over the entire sequence. (c, d) Mutations in the 4 DLBCL studies (Table S2) in ACTB (c) and ACTG1 (d) are mostly concentrated in the N-terminal half of the sequence. (e, f) Mutations in the multiple myeloma study (Table S2) in ACTB (E) andACTG1 (f) are located at the N-termini. Green bullets are missense mutations, brown bullets are in frame mutations and black bullets are truncating mutations. Data derived from the Mutations tab.
